# A New HLA-Based Distributed Control Architecture for Agricultural Teams of Robots in Hybrid Applications with Real and Simulated Devices or Environments

**DOI:** 10.3390/s110404385

**Published:** 2011-04-14

**Authors:** Patricio Nebot, Joaquín Torres-Sospedra, Rafael J. Martínez

**Affiliations:** 1 Department of Engineering and Computer Science, Universitat Jaume I, Avda. Sos Baynat S/N, E-12071, Castellón, Spain; E-Mail: jtorres@uji.es; 2 LSyM - IRTIC Institute, University of Valencia, C/Catedrático José Beltrán 2, 46980, Paterna, Spain; E-Mail: rafael.martinez@uv.es

**Keywords:** hybrid systems, improved simulation system, agricultural robotics, control architecture, HLA-based system

## Abstract

The control architecture is one of the most important part of agricultural robotics and other robotic systems. Furthermore its importance increases when the system involves a group of heterogeneous robots that should cooperate to achieve a global goal. A new control architecture is introduced in this paper for groups of robots in charge of doing maintenance tasks in agricultural environments. Some important features such as scalability, code reuse, hardware abstraction and data distribution have been considered in the design of the new architecture. Furthermore, coordination and cooperation among the different elements in the system is allowed in the proposed control system. By integrating a network oriented device server *Player*, Java Agent Development Framework (*JADE*) and High Level Architecture (*HLA*), the previous concepts have been considered in the new architecture presented in this paper. *HLA* can be considered the most important part because it not only allows the data distribution and implicit communication among the parts of the system but also allows to simultaneously operate with simulated and real entities, thus allowing the use of hybrid systems in the development of applications.

## 1. Introduction

The use of robotic applications in forestry or agriculture is widespread. This paper will deal with the agricultural robotic systems which work into orange groves. These systems should perform some general applications, such as navigation, and specific maintenance tasks. In orange groves, the robotic system can be used to collect oranges, detect illnesses, eliminate weeds, fertilize, or simply explore the grove to eliminate rests of trees by the pruning.

In the bibliography, the description of cooperative systems, composed by some different robots, for agricultural tasks is not common. In general, a single complex robot which performs specific actions on the environment is commonly suggested in the literature [1–5].

The control architecture is one of the most important parts to develop in a robotic system, especially if it is composed by some robots which must cooperate. This control architecture must give support for all the facilities of the system and forms the backbone of the robotic system. The right choice of the architecture can facilitate the specification, implementation and validation of the applications implemented for the system. In the literature, we can find some important architectures which are able to be used in our cooperative system.

- **Robot Operating System** (*ROS*): It is a framework for robot software development, providing operating system-like functionality on top of a heterogeneous computer cluster. This framework was originally developed in 2007 by the Stanford Artificial Intelligence Laboratory in support of the *Stanford AI Robot* project under the name *switchyard*. Although it is intended to be cross-platform, it is only fully supported by a few Linux distributions.
- **Orca/Orocos:** *Orca* is another open-source framework, released under *LGPL* and *GPL* licenses, for developing component-based robotic systems. It was initially a part of an EU sponsored project, *OROCOS 2002*, but it was renamed to *ORCA*. This framework is based on *CORBA* and it provides the means for defining and developing the parts of complex robotic systems.
- **Umbra:** *Umbra* is a framework for modeling and simulation. This framework allows generating models and simulations for intelligent system development, analysis, experimentation, and control and supports the analysis of complex robotic systems. The models in Umbra include 3D geometry and physics of robots and environments. Model components can be built with varying levels of fidelity, so the models built with low fidelity for conceptual analysis can be gradually converted to high fidelity models for later phase detailed analysis. Within control environments, the models can be easily replaced with actual control elements.

Abstraction of the hardware, code reuse, scalability and other features are included in these three alternatives. Moreover, some of these architectures implement the capability to manage the possible coordination or cooperation among different entities that could be involved in the system to perform the different tasks. Additionally, the capacity of allowing communication, implicit or explicit, between the elements in the system is also implemented by some of them.

Table 1 introduces a comparison considering all these control architectures and the proposed *Agriture*. Important and relevant measurements have been included in this comparison.

**Figure 1.**
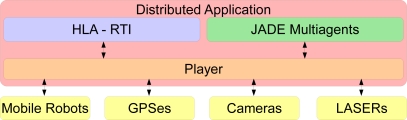
The new distributed HLA-based architecture.

The research described in this paper is focused on the development of a cooperative system to control a team of mobile robots. The intention is to use the proposed system in agricultural environments developing different maintenance tasks. As mentioned before, in any robotic system it is necessary to choose a control architecture to support the system. However, none of the architectures reviewed before fit well in our cooperative system requirements. In our case, including the capabilities of the other architectures, it is also desirable to have the architecture based on international standards and allow simulations of the elements in the system in order to deploy or debug the applications without the necessity to move to the outdoor scenario. These concepts are not present in the cited architectures. For this reason we introduce a new control architecture called “*AGRIcultural architecTURE*” (*Agriture*), which integrates three different systems (*Player* [9], *JADE* [10] and *HLA* [11–13]) that interact to provide a solid control architecture for developing cooperative robotic system. Figure 1 introduces the basic description of a distributed application for the proposed architecture.

The rest of the paper is organized as follows. Firstly, the design and implementation of the proposed architecture, *Agriture*, is introduced in Sections 2 and 3. In Section 4, some distributed applications which can use *Agriture* are detailed. Finally, the conclusions and some future research lines are presented.

## 2. Design of the *Agriture* Architecure

As mentioned, the purpose of this work is to develop a system able to control a team of mobile robots in agricultural environments. This system is based on the *Agriture* architecture which is the backbone for the system. *Agriture*, as explained, is based in three subsystems: Player, JADE and HLA, each giving different features to the architecture.

The most interesting system added to the architecture is the *HLA* (*High Level Architecture*) subsystem. *HLA* provides a framework to create complex simulations using simple components and is defined under *IEEE Standard 1516–2010*. There following institutions use *HLA* for their applications:

- **NASA:** This agency uses *HLA* for integrating numerous specialized simulators into larger overall space mission simulations [14]. It is also used for formation flying using multiple spacecraft and ground stations. Furthermore, some applications related to modeling natural Earth or planetary geophysical systems are based on *HLA*.
- **Boeing:** Linking Boeing’s Joint Strike Fighter (JSF) full-mission simulator with U.S. Air Force Air Combat Command simulators off-site in order to conduct real-time cooperative training missions [15].
- ***HLA*** **in robotics:** In [16,17] a new distributed simulation environment, Symbricator3D, is introduced to simulate robot swarms as well as modular robots. Its underlying framework is the game and simulation engine “Delta-3D”, which also supports the *HLA* standard for providing distributed simulation. In [18] a new multidisciplinary modeling and collaborative simulation system based on HLA related to multi-robot systems is developed.

**Figure 2.**
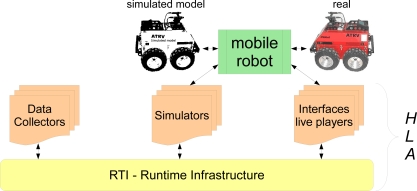
HLA offers the possibility to employ real and simulated entities at the same time.

HLA simulations are divided into federates. “A *HLA* federate can be a computer simulation, a manned simulator, a supporting utility (such as a viewer or data collector), or even an interface to a live player or instrumented range” [19]. This property of the HLA systems opens a very big field of applications. It allows the use of both real and simulated entities at the same time, being these entities robots, environments, or any part of the system able to be simulated. This is specially useful when the system is optimized for a specific application, like the case of agricultural robotics. The relationship among the different parts is depicted in Figure 2.

In the previous figure, an example of abstraction of the mobile robot is used. Depending on the application, a real or simulated instance of the mobile robot (*ATRV-2*) is used to perform the corresponding tasks.

The new architecture, shown in Figure 1, is composed of three layers. The physical layer is related to all the real or simulated devices (robots, cameras, sensors, ...). They are the agents which interact with the real or simulated environment. One important feature is that each of these elements has its own specifications and communication protocols.

The architecture middleware implements: (1) the cooperation and coordination tasks, (2) communication between the elements, and (3) abstraction of the external devices (elements). This middleware involves three parts. One of them is in charge of implicit communication and establishes the links and data exchange format between the different elements; another part is related to explicit communication and takes charge of determining which information is transmitted and how the tasks are assigned to physical elements; the last one provides machine independence to the system because each robotic system is abstracted. The system does not work directly with the real robots, and their abstraction allows to use basic commands to control the robots. In this way, the robot can be easily replaced by a different robot without having to recode the middleware. Only the abstraction module related to the robot has to be replaced.

Finally, the highest part of our architecture is composed by the distributed applications. These distributed applications are controlled by the middleware layer, which is in charge of assigning the different tasks to each specific element or device. When an application is tested and it correctly works, it can be added to the middleware as a part of the whole system. This is a feature of *Software Scalability*.

**Figure 3.**
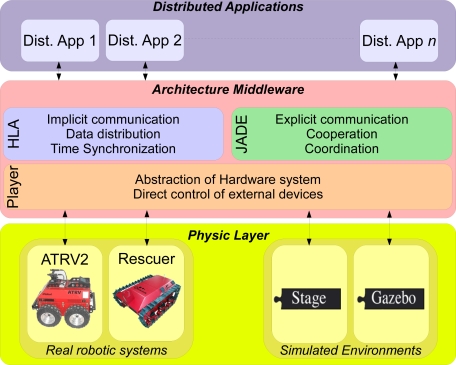
The new distributed HLA-based architecture.

The whole system, with the three layers detailed, can be seen in Figure 3.

**Figure 4.**
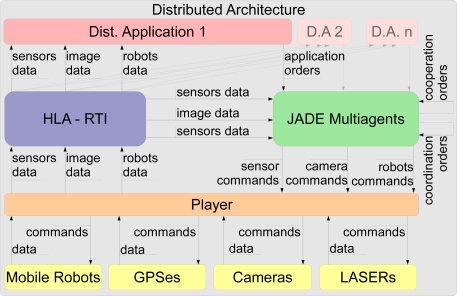
Flow chart.

The diagram shown in Figure 4 represents how the signals flow in the proposed architecture. In this chart, an instance of device (*Camera*, *LASER*, *GPS* or *Robot*) is directly linked to a real or a simulated element.

## 3. Implementation of the *Agriture* Architecture

### 3.1. Physical Devices/Hardware

**Figure 5.**
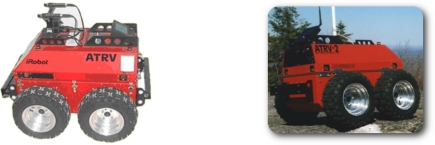
ATRV-2 robotic system.

**Figure 6.**
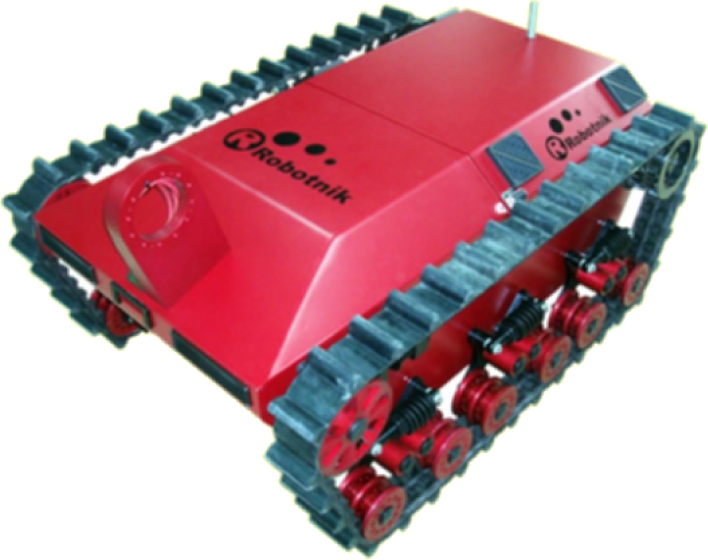
Rescuer robotic system.

**Figure 7.**
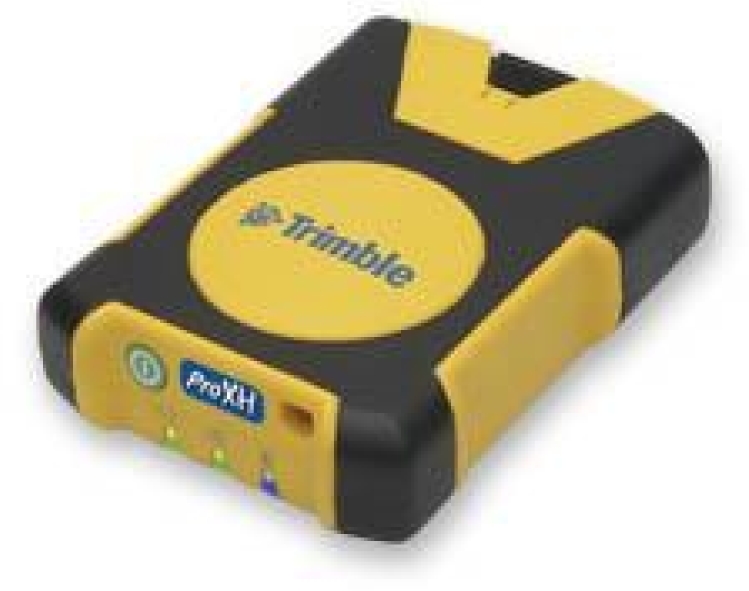
GPS—Pathfinder ProXH receiver.

**Figure 8.**
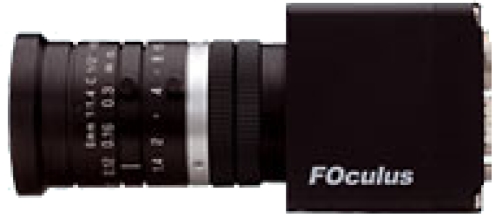
Vision System—FOculus 124IC with auto iris lens.

**Figure 9.**
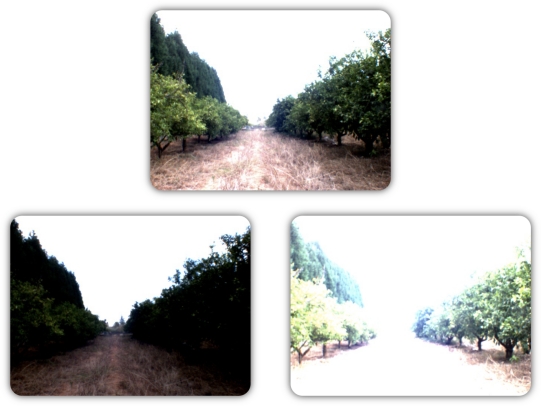
Differences in the same capture with different apertures of the iris.

**Figure 10.**
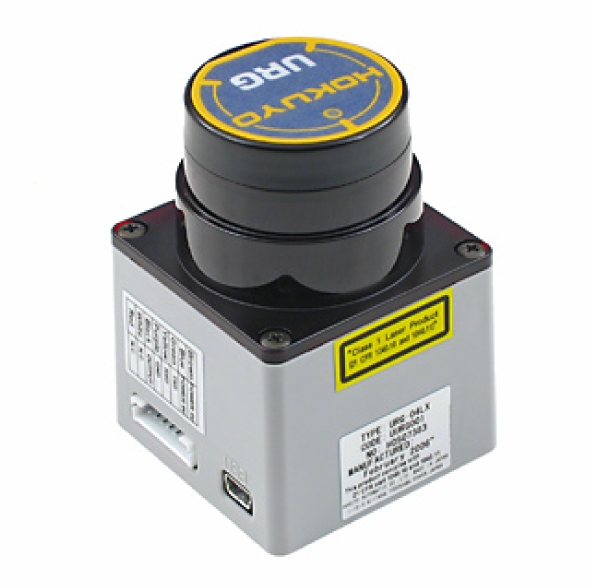
LASER - HOKUYO’s LASER.

**Figure 11.**
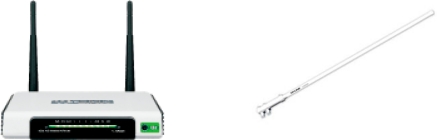
TP-LINK WiFi router and antenna.

The physical layer of the architecture contains all the hardware elements of our system. They are directly managed by *Player*. In the experiments on outdoor environments, such as in Agricultural Robotics, the following devices will be used:

- **ATRV-2:** The *ATRV-2*, shown in Figure 5, is a rugged four-wheel drive, differentially steered all-terrain robot vehicle for outdoor robotic research and application development. It is stable in wide varied terrains and it can traverse them easily. All its features make the *ATRV-2* a good alternative for use in outdoor environments such as the orange groves. The ATRV-2 Provides:
  – An internal PC computer with data and power ports for user hardware additions.
  – Full front sonar coverage, with 4 side and 2 rear sonars.
  – Mobility object-oriented software development environment.
  – Run Time: 4.5 h average.
  – Translate Speed: 2 meters per second.
  – Payload: 50 kg user added equipment.
- **RESCUER:** It is a mobile platform extremely solid and very appropriate for outdoor environments, even hazardous to access (see Figure 6). It is appropriate for scientific research. The researchers can add components (cameras, sensors, hydraulic arms or grippers) due to its modular system. A big quantity of equipment can be added whether over its platform or inside it.
  Other features of this robot are:
  – Dimensions: 1,100 × 780 × 600 mm.
  – Payload: 200 kg.
  – Speed: 1.25 m/s.
  – Software architecture based in player/stage.
  – Autonomy: 8 h in normal operation
- **GPS:** Each robot has its own *GPS* module, concretely a *GPS Pathfinder ProXT Receiver*. This device is composed of a sub-meter precision GPS receiver, an antenna, and an all-day battery as can be seen in Figure 7. Moreover, it is totally cable-free, rugged and weatherproof, so it suitable for outdoor environments such as orange groves.
  The use of GPS is essential for localization and navigation of the robotic system in orange groves, as it provides the global position of the robot. This particular GPS sensor is a “differential GPS”, which offers greater precision than other standard GPS receivers, and they can also make use of the DGPS Spanish Network.
- **Vision system:** The vision system is composed by a *VGA* camera with *CCD* image sensor and auto iris control. Concretely, the *FOculus FO124IC* (Figure 8) has been used after testing some other alternatives. The features of the camera are:
  – 1/3′ IT CCD image sensor.
  – Color camera.
  – Progressive Scan mode.
  – 60 fps, 640 (H) × 494 (V), External Trigger.
  – Auto IRIS Lens Control.
  – Firewire - IEEE 1394.
  The lens used is a Pentax H612E(HK) lens which has 0.5′ format, 6.0 mm focal length and its iris is automatically controlled by the camera.
  This vision system provides *VGA* color images whose resolution is enough for further applications. Furthermore, it allows the auto iris control which is necessary in outdoor scenarios such as orange groves. In the next figure, Figure 9, three different captures have been shot with a similar FOculus camera without the auto iris control. The upper image is related to a capture where the aperture of the iris has been optimized for the lighting conditions. However, the image obtained may not adequately capture the elements of the grove if the iris is not properly set (the images located at the bottom left and bottom right).
  In these captures, it can be seen that the bottom left image is so dark that the elements can not be distinguished. In the bottom right image, the iris is too open and this produces a “burn” image where most of the elements have highest brightness possible.
- **LASER detector:** To perform the detection of the obstacles located in front of a robot, a *HOKUYO’s LASER* system, shown in Figure 10, has been chosen. This detector provides a field-of-view around 240 degrees and its angular resolution is close to 36 degrees with a scanning refresh rate of up to 10 Hz.
  Due to the problems related with outdoor environments and this class of sensors, only the objects located near the robot will be detected.
- **WiFi Network:** This system is used to allow the wireless communication between the different robots. This network should allow the communication among all the robots in any place of the grove. Moreover, it should provide Internet connectivity in order to obtain, for instance, satellite images.
  For this reason a *TP-Link* 3G wireless router (TL-MR3420) has been used (Figure 11). This router creates a wireless network under the standard *IEEE 802.11N*. Moreover, it allows 3G connectivity via a USB 3G stick. This router is placed in the grove besides the human operator and its position is kept unchanged.
  This kind of networks, based on standard *IEEE 802.11N*, provides better coverage and speed than traditional wireless networks (*IEEE 802.11g*). Moreover, two 1.5 meter omnidirectional 15dbi antennas (TP-Link TL-ANT2415D also shown in Figure 11) are connected to the router to have, approximately, a coverage of one kilometer radius.

Example of the Information Provided by the Physical Devices

**Figure 12.**
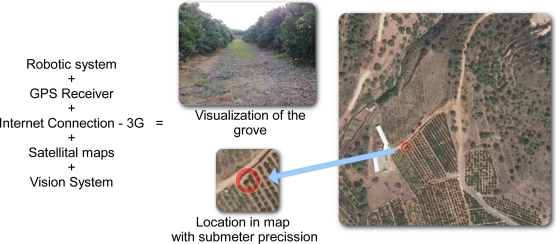
Example of the visual data collected by all the sensors.

With all the “sensors” previously listed, a global view of the real environment can be obtained. On the one hand, the vision system and *LASER* provide local information related to the real environment in real time. On the other hand, the *GPS* module sends the coordinates to a maps service via Internet (WiFi router with 3G connection) that allows the robot to obtain the image of the area where it is placed. A graphical description is shown in Figure 12.

According to the previous figure, each robot has visual information about the path it is navigating (provided by the vision system) and it can extract some extra information about its position and trajectory from the satellite image. Moreover, any other robot of the system will provide similar information about its localization. All the robots can share this information in order to coordinate efficiently one or more tasks. The special WiFi network will allow the high speed communication among all the robots of the system.

### 3.2. The Middleware Layer

In this subsection, the three different systems which forms the middleware layer are described.

- **JADE:** JADE is a software framework designed to develop agent-based applications in compliance with the *FIPA* specifications for interoperable intelligent multiagent systems. It is a software framework fully implemented in Java language and simplifies the implementation of multiagent systems through a middleware and through a set of graphical tools that supports the debugging and deployment phases [20].
  The *JADE* middleware implements an agent platform for execution and a development framework. Also, it provides some agent facilities such as life cycle management, naming service, message transport and parsing service, and a library of *FIPA* interaction protocols ready to be used [20].
- **The High Level Architecture:** *HLA* provides a general framework in which the researchers can structure and describe their final applications. In any case, *HLA* is neither a simulator nor a modeling tool. Furthermore, *HLA* does not generate data or simulate because it does not eliminate programming.
  The main objective of *HLA* is to generate systems (*Federations*) based on *reusable* components of different nature (*Federates*) which can *interact* among them easily through a distributed, real-time operating system. To perform this task, there are three main components in the *HLA* architecture:
  – A set of rules: *HLA* consists of a set of ten rules which must be obeyed in order to govern the overall system and to govern each participating component. Five of them defy the operations of the federate and the other five are related to the federations.
  – *An Object Model Template:* The *Object Model Template*, *OMT*, provides a standard for defining and documenting the form, type and structure of the information shared within a simulation. This model defines the *Federation Object Model* (information shared for each federation), the *Simulation Object Model* (internal operations of each federate that can be used externally) and the *Management Object Model* (identification of the objects/interactions used to manage a federation and introduction of universal definition).
  – *Interface Specifications:* The *Interface Specifications*, *IS*, describes the runtime services between each federate and the *RTI*. There are six classes of services. The *HLA Interface Specification* defines the way these services are accessed in an application programmer’s interface (*API*).
- **Player/Stage:** One of the most widely used software nowadays for programming multirobot applications is the *Player/Stage* project [9]. *Player* is a network oriented device server that provides clients with network-oriented programming interfaces to access actuators and sensors of a robot. It employs a one-to-many client/server style architecture where one server serves all the clients of a robot’s devices and it relies on a TCP-based protocol to handle communication between client and server.
  Accompanying *Player* is the robot simulator Stage, a lightweight, highly configurable robot simulator that supports large populations of robots and allows programmers to control these virtual robots navigating inside a virtual environment.

In the system, each one of the subsystems is in charge of a main task. In that way, the coordination and cooperation task, as well as the explicit communications, rely on *JADE*, whereas the implicit communication, the data distribution and the control of the simulated entities are assigned to *HLA*. Finally, *Player* is in charge of the hardware abstraction and the control of the real (or simulated) entities in the system.

## 4. Possible Distributed Applications of *Agriture*

The proposed architecture, *Agriture*, can interact with real and simulated devices. Depending on the final application, a concrete device (real or simulated) can be used. This can be useful in the procedure used to set or optimize some parameters of the whole system.

**Figure 13.**
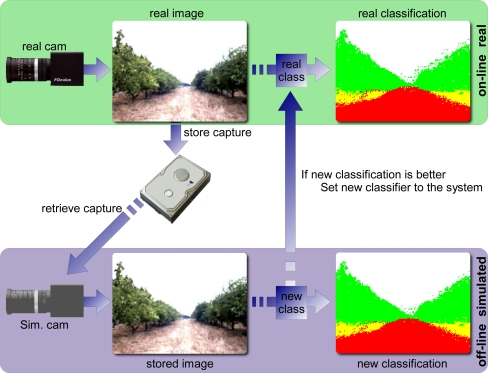
Offline application: HLA allows to simulate all the elements to optimize part of the system.

As an example, this system can be run in a real orange where a classifier processes the images obtained by the vision system in order to identify the elements of the grove as depicted in Figure 13. The sequence of images captured can be stored for further research or debugging tasks. Although the classifier used can be good enough, its performance can be improved by setting better its parameters. In this case, an off-line simulation can be run using the stored data. It means that all the elements and devices of the system are simulated but they provide real data from the previous real run. In the figure, it can be seen that the virtual camera provides a capture done in the real orange grove to the simulated environment. In the simulation, other classification alternatives (using the same classifier with different parameter or choosing different base classifiers) can be tested and the original classifier (the one used in the real runs) can be replaced if it is outperformed by another alternative.

In the previous image, it can be seen that the classification obtained in the real run is good. However, there are some misclassifications. Some of them are near the boundaries between the sky (white in classification image) and orange crowns (denoted with green pixels). In the experiments done off-line, a better classifier has been used with the same purpose and it provides slightly better results. Although both classifications are similar (close to 95% of correctly classified elements), the second one is better because the misclassifications near the boundaries tend to disappear.

Although the use of off-line runs have been introduced to optimize a task of the real system (a classification task in the example), it can be used to evaluate a new task (not implemented in the real system) which uses the information provided by the sensors used in the real system. This application involves using a completely simulated environment with virtual devices to test it. These simulations with real data can be useful when the number of alternatives considered for a new task are high because they can be tested under the same experimental framework (the same real data) without using the real devices.

Furthermore, the use of *Agriture* can be considered for hybrid applications in which real and simulated elements are used together. In hybrid applications, both kinds of elements (real and virtual) must be present.

**Figure 14.**
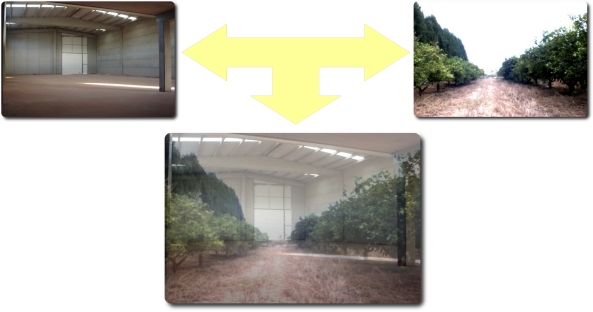
First application: HLA offers the possibility to virtualize an orange grove into a warehouse.

In a first hybrid application, the real robots move in a free-obstacle environment (an example is shown in Figure 14). The orange grove can be simulated but the interaction and navigation of the different robots can be real. For instance, the robots can be located into a warehouse but the information provided by the camera and GPS can be simulated to make the robot believe it is navigating in a real grove. In this application, the robot’s behavior in an orange grove can be tested without the robot being there. In this case, the real robots will navigate in the simulated grove without the risk of damaging the environment. Moreover, the economical costs of optimizing the system (testing different algorithms for a task) are reduced because the outdoor field experiments are avoided.

**Figure 15.**
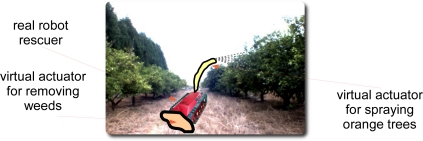
Application 2: HLA offers the possibility to virtualize an element/device of a real robot.

The second hybrid application considered is depicted in Figure 15. This application consists in using both real and virtual devices in a real environment (orange grove). In this system, a real robot could perform a virtual task in parallel with other real tasks. For instance, the orange trees can be virtually sprayed or the weeds located in the path can be virtually removed while the robot is navigating through the grove. In this way, some different algorithms to perform a new task can be tested while the robot is running another real application. The real devices, such as cameras and other sensors can provide real information to the new virtual element, so the simulation can be more realistic. It can be also useful in the case in which the availability for executions of the real devices (robots and sensors) into a simulated environment (such as the warehouse previously mentioned) is low, because the resources are required to perform a real application. In this case, the new virtual applications can be tested *in situ* while the required real task is being done.

## 5. Conclusions

In this paper, *Agriture*, a new architecture to implement an agricultural multirobotic system has been introduced. In *Agriture*, *Player/Stage* is in charge of the hardware (real or simulated) abstraction. The coordination and cooperation tasks rely on *JADE* whereas the implicit communication is assigned to *HLA*. They have been selected to improve the *reusability*, *scalability* and *interoperability* of system.

Furthermore, the use of *Agriture* has been introduced either in real or simulated environments. It can be used in real applications or it can be applied to simulate experiments. In addition, the proposed architecture can be used in hybrid systems in which real and simulated elements (devices and environments) can interact.

## Figures and Tables

**Table 1.** Comparison among different architectures.

	
	**ROS**	**ORCA/OROCOS**	**UMBRA**	**AGRITURE**
*Cross-platform*	×[6]	✓	✓	✓
*Real-world interaction*	✓	✓	*×*	✓
*Hardware abstraction*	✓	✓	✓	✓
*Code reuse*	✓	✓	✓	✓
*Distributed Multi-robot*	✓	✓	✓	✓
*Free license*	✓	✓	*×*	✓
*Efficient distributed operation*	*×* [7]	×[8]	✓	✓
*Scalability*	*×* [7]	✓	✓	✓
*Hybrid systems*	*×*	*×*	*×*	✓
